# Cutaneous leishmaniasis in a severely immunocompromised HIV patient in Kumbo, Northwest region of Cameroon: case report

**DOI:** 10.1186/s13104-017-2751-1

**Published:** 2017-08-25

**Authors:** Larry N. Tangie, A. Desmond, Leopold N. Aminde, Annabel M. Ako, P. M. Halle

**Affiliations:** 1 Banso Baptist Hospital, Kumbo, Cameroon; 2 Nkwen Baptist Hospital, Bamenda, Cameroon; 3 School of Public Health, University of Queensland, Brisbane, Australia; 4 Faculty of Medicine, University of Douala, Douala, Cameroon; 5 Department of Internal Medicine, Douala General Hospital, Douala, Cameroon

**Keywords:** Cutaneous leishmaniasis, HIV, Cameroon, Case report

## Abstract

**Background:**

Leishmaniasis is a rising opportunistic infection in individuals with human immunodeficiency virus (HIV). Cases of leishmania and HIV co-infection have been documented in several countries in the world with most reporting on the association between visceral leishmaniasis (VL) and HIV. We herein report the case of cutaneous leishmaniasis (CL) occurring in an HIV seropositive patient.

**Case presentation:**

A 28 year old Cameroonian female diagnosed with HIV for 6 months earlier, presented to our facility with a 3 months history of non-painful rash. Clinical examination revealed non prurigeneous papulo-nodular lesions on the face and thighs which later became crusty ulcerative lesions. Giemsa staining with examination under oil objective immersion identified amastigotes and a diagnosis of CL was made which was managed with amphotericine B (1 mg/kg of body weight) for 14 days with mild improvement of lesions. Patient developed hypokalemia due to the amphotericine B during admission which was corrected and died 1 month after discharge.

**Conclusions:**

Current evidence suggest higher incidence of VL in HIV, however we report the occurrence of CL in HIV. A high index of suspicion for CL is warranted among clinicians in Africa when faced with HIV patients with inconsistent cutaneous rash.

## Background

Cutaneous leishmaniasis (CL) is caused by obligate intracellular protozoa of the genus leishmania and transmitted by the sand-fly, *Phlebotomus* [1]. CL is one of the four different forms of leishmaniasis; the other three being visceral leishmaniasis (VL) or (Kala-azar), mucocutaneous leishmaniasis and diffuse mucocutaneous leishmaniasis [2].

Cases of leishmania and HIV co-infection have been reported from 35 countries around the world. Most of these studies report an association of VL and HIV and were carried out mainly in southwestern Europe [3]. There are few reports of a co-occurrence of CL with HIV in sub-Saharan Africa. The few cases documented are mainly from the western part of the continent including Burkina Faso, Mali and Senegal [4–7]. Both CL and VL have previously been reported to be endemic in the Northern region of Cameroon [8, 9]. Here we report a case of cutaneous leishmaniasis and HIV co-infection in a patient from the Northwest region of Cameroon.

## Case presentation

A 28 year old female housewife from Kumbo, a rural area in the North West region of Cameroon, Sub Saharan Africa, who presented to our facility in October 2015 with a 3 months history of non-painful rash. The rash was of gradual onset, progressive non itchy and occurred on areas of her face and thighs. It later evolved to crusty ulcerative lesions prompting consultation in our unit.

She was diagnosed positive for HIV 6 months prior to consultation with a CD4 count of 10 cells/µl and was initiated on highly active antiretroviral therapy (HAART) Tenofovir, Lamivudine and Efavirenz. There was no history of intravenous drug use (IVDU).

**Fig. 1:**
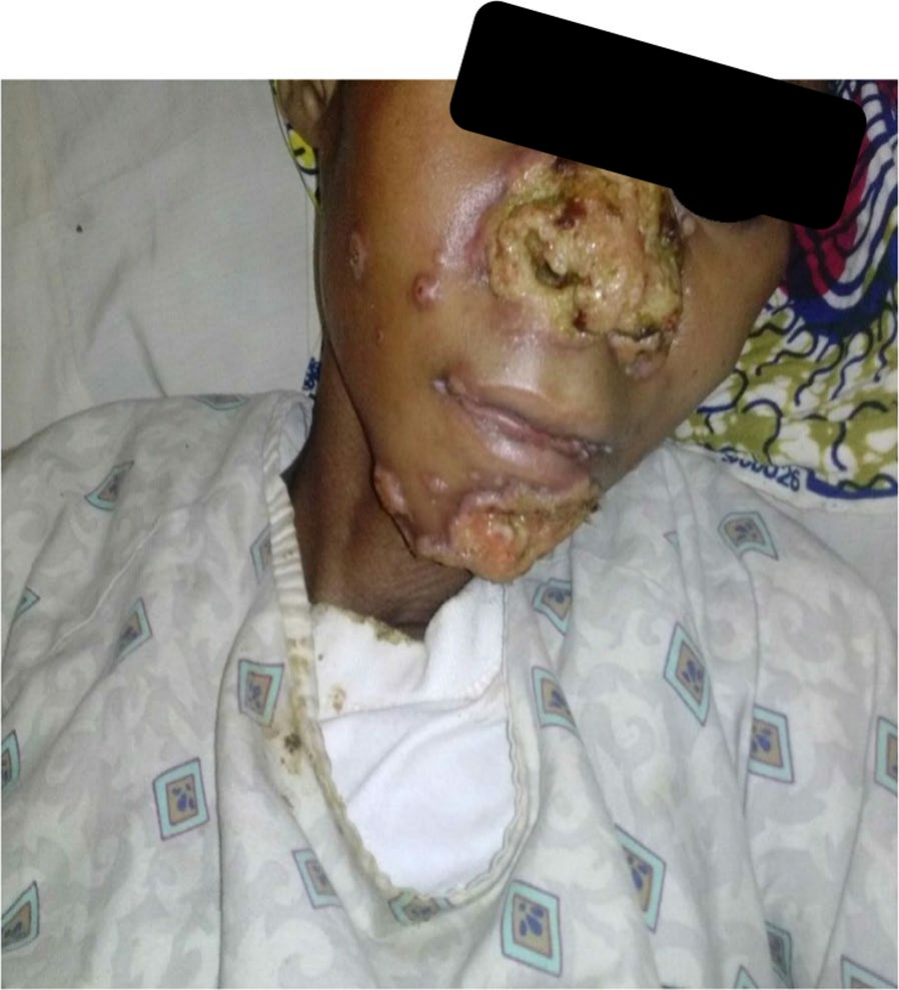
Nasal and mental area ulcerations

**Fig. 2:**
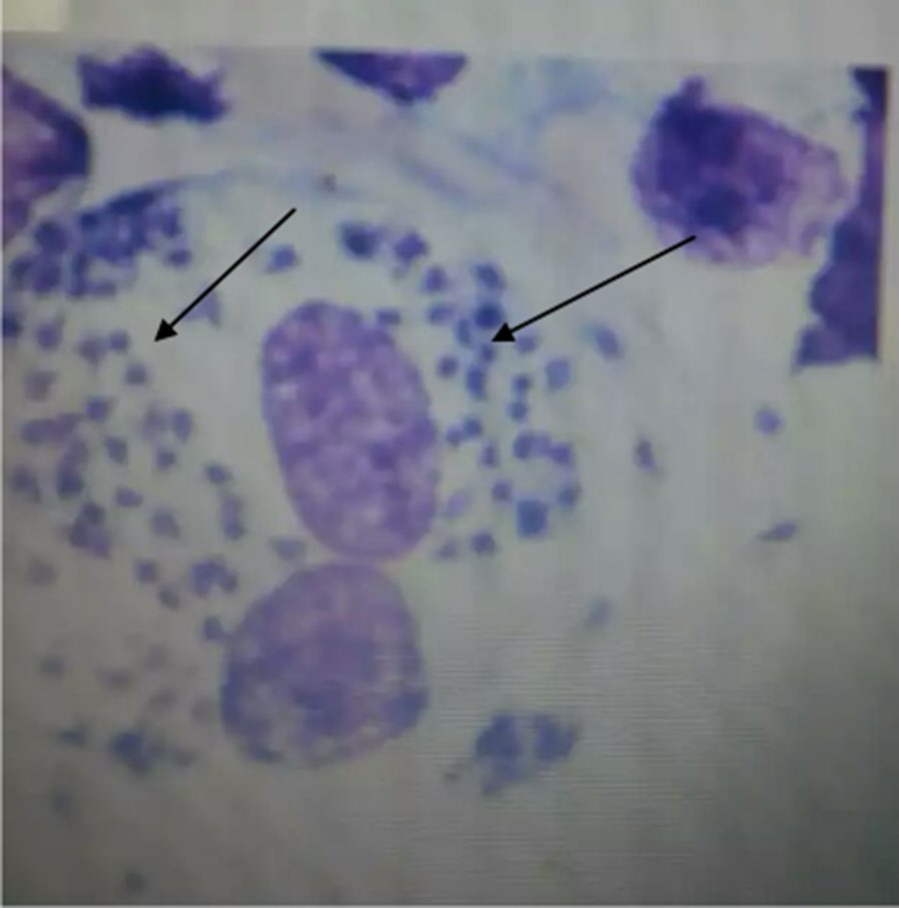
Microscopy showing amastigotes

On physical examination, she was cachectic and ill looking with a BMI of 15.6 kg/m^2^, with non-tender crusty ulcerated lesions around the nasal area (with destruction of nasal tissue and nasal septum), non-tender ulcerations around the mental regions with a few papular lesions around the cheeks (Fig. 1). No mucosal lesions were found in the mouth, there were neither lymphadenopathy, nor hepatomegaly and splenomegaly on abdominal palpation. She had spaced non ulcerative papular lesions on her thighs. There was neither hypoesthesia nor any nerve involvement around the ulcerations. The rest of physical exams was unremarkable. A working diagnosis of cutaneous leishmaniasis was made with a differential of tertiary syphilis, cutaneous mycosis and cutaneous tuberculosis. The TPHA/VDRL done was non-reactive while a biopsy of ulcerations was negative for acid fast bacilli (AFB). A skin scrape of the lesions was negative for KOH while absence of hypoesthesia and a negative biopsy for AFB helped to exclude lepromatous leprosy. Venous blood was collected in an ethylenediaminetetraacetic acid (EDTA) tube, spinned at 3000 rpm for 5 min. A sample from the Buffy coat was obtained and a thin film made. The slide was stained with Giemsa and examined with oil objective immersion to identify the amastigote (Fig. 2).

She was treated with amphotericine B given intravenously at 1 mg/kg for 2 weeks with mild improvement in lesions. Renal function test was normal before and 1 week after initiation of amphotericine B. She developed hypokalemia due to amphotericine B which was corrected with intravenous potassium. She was discharged but died 1 month later.

## Discussions

Infections caused by the protozoan leishmania represent a wide range of diseases, including CL, mucocutaneous leishmaniasis (ML), and visceral leishmaniasis (VL) [10]. The disease is endemic in 88 countries of southern Europe, Central and South America, Africa, the Middle East and the Indian subcontinent. More than 350 million men, women and children are at risk of leishmaniasis worldwide [11]. The are 12 million individuals suffering from the disease worldwide, with half a million new cases of VL and 1.5 million cases of CL registered annually [11]. The incidence of infections caused by leishmania in HIV-infected patients is increasing throughout the world [12].

Although VL is the most frequent form of leishmania infection seen in HIV-infected patients, CL and HIV co-infections are also becoming frequent in these individuals [12]. CL manifests as single or multiple papules, nodules or plaques, which are non-itchy, painless and with or without ulceration, usually over exposed areas of the body [8]. Both diseases (leishmaniasis and HIV) infect and multiply in monocytes, and in the case of a co-infection, there is a mutual replication of both in host cells. This may further lower the body’s defense mechanism with resultant poor prognosis as was evident in our patient.

In fact, a leishmaniasis infecting myeloid cell promotes HIV replication while HIV in turn, promotes an uptake of leishmaniasis by macrophages and amplifies parasitic replication in monocytes [12]. In cases of leishmania and HIV co-infection, CD4 <200cells/µl and malnutrition could lead to reactivation of latent infections (reactivation leishmaniasis).

Our case correlates with earlier reports that leishmaniasis tends to occur in advanced stages of HIV infection [11]. Considering the very low CD4 count, and absence of other opportunistic infections in our case suggests that CL could be a potential opportunistic infection in HIV. Interestingly despite the low CD4 count there was no visceralization of the leishmania species. Although data suggest a higher incidence of VL in HIV positive patients, more cases of CL are being reported in HIV positive patients [7, 10, 12]. The Giemsa staining used in our setting for diagnosis of leishmaniasis is rarely done in most remote primary health care facilities as most staff are unskilled and even more the absence of necessary equipment. The standard therapy for CL is antimoniate, however other second line drugs such as amphotericine B and ketoconazole are being used. Poor response to therapy could be secondary to severe immunodepression and suboptimal therapy. Currently, the standardized treatment of CL for immunocompetent patients is either intralesional or systemic antimonials, and HIV-infected patients have also shown a good response to antimonials [13]. Liposomal amphotericine B has also been found to be effective [14]. The use of non liposomal amphotericine B intravenously due to non-availability of the liposomal form in our setting could also explain the poor response to therapy.

## Conclusions

Leishmaniasis is a rising opportunistic infection in HIV. Though data suggest more cases of VL in HIV, there are increasingly cases of CL being reported in HIV positive patients as demonstrated by the above report. Appropriately investigating suspicious cutaneous lesions in HIV positive patients may lead to further detection. We therefore recommend a high index of suspicion amongst clinicians when faced with an atypical rash in an HIV positive patient.

## Abbreviations

CL: cutaneous leishmaniasis
HIV: human immune deficiency virus
HAART: highly active antiretroviral therapy
IVDU: intravaneous drug use
VL: visceral leishmaniasis
